# E-cigarette aerosol exposure can cause craniofacial defects in *Xenopus laevis* embryos and mammalian neural crest cells

**DOI:** 10.1371/journal.pone.0185729

**Published:** 2017-09-28

**Authors:** Allyson E. Kennedy, Suraj Kandalam, Rene Olivares-Navarrete, Amanda J. G. Dickinson

**Affiliations:** 1 Virginia Commonwealth University, Department of Biology, Richmond, VA, United States of America; 2 Virginia Commonwealth University, Department of Biomedical Engineering, Richmond, VA, United States of America; University of Colorado Boulder, UNITED STATES

## Abstract

Since electronic cigarette (ECIG) introduction to American markets in 2007, vaping has surged in popularity. Many, including women of reproductive age, also believe that ECIG use is safer than traditional tobacco cigarettes and is not hazardous when pregnant. However, there are few studies investigating the effects of ECIG exposure on the developing embryo and nothing is known about potential effects on craniofacial development. Therefore, we have tested the effects of several aerosolized e-cigarette liquids (e-cigAM) in an in vivo craniofacial model, *Xenopus laevis*, as well as a mammalian neural crest cell line. Results demonstrate that e-cigAM exposure during embryonic development induces a variety of defects, including median facial clefts and midface hypoplasia in two of e-cigAMs tested e-cigAMs. Detailed quantitative analyses of the facial morphology revealed that nicotine is not the main factor in inducing craniofacial defects, but can exacerbate the effects of the other e-liquid components. Additionally, while two different e-cigAMs can have very similar consequences on facial appearances, there are subtle differences that could be due to the differences in e-cigAM components. Further assessment of embryos exposed to these particular e-cigAMs revealed cranial cartilage and muscle defects and a reduction in the blood supply to the face. Finally, the expression of markers for vascular and cartilage differentiation was reduced in a mammalian neural crest cell line corroborating the in vivo effects. Our work is the first to show that ECIG use could pose a potential hazard to the developing embryo and cause craniofacial birth defects. This emphasizes the need for more testing and regulation of this new popular product.

## Introduction

E-cigarettes (ECIGs) are battery-powered nicotine delivery systems that are advertised as a safe alternative to tobacco smoking [1–3]. The ECIG device contains a reservoir filled with an e-liquid that is heated by an atomizer to produce an aerosol that is inhaled by the user. These e-liquids are comprised of nicotine, flavor additives, and compounds that aid in delivery [4]. While ECIGs were initially marketed as a tobacco cessation tool, they have since become a global trend for recreational use. The number of young people using ECIGs has increased by almost 10 -fold over the past five years [5], and the rates of ECIG use among women in the USA are also substantially increasing [6]. Most concerning is that some pregnant women use ECIGs [7] [8], believing that they are safer than traditional tobacco cigarettes [7, 9] and only associated with minor health hazards [10]. However, little is known about the effects of ECIG aerosol mixtures (e-cigAM) on human health, and even less is known about the potential impact on the developing embryo.

It is well established that traditional cigarette smoking can increase the risk for craniofacial defects such as cleft palate [11–17]. Based on studies in animal models, at least some of these defects can be attributed to the teratogenic effects of the chemicals that are present in cigarette smoke [18–21]. On the other hand, it is less clear whether other chemicals produced by ECIG use could also perturb facial development.

The main components of e-liquids are nicotine, propylene glycol, vegetable glycerin, water and many different flavor additives, [4]. The federal government has only just recently begun to regulate the composition of these e-liquids [22], in part due to the inaccuracy in ingredients reported by manufacturers and their unknown effects on human health [5, 23, 24]. For example, the heating process of propylene glycol can produce toxicants such as acrolein, formaldehyde, and benzene, while glycerin can decompose to form reactive aldehydes [25, 26] [27–29]. Additional compounds such as hemiformals, glycidol, dihydroxyacetone, and vinyl alcohol isomers have been found in chemical analysis of ECIG aerosols, all of which can have adverse human health effects [30]. Even more concerning is the endless numbers of flavor compounds in e-liquids, many of which are aromatic aldehydes such as benzaldehyde and vanillin [31]. While the effects of these aldehydes on their own are not clear, oxidation upon heating can convert them to benzoic acid and benzene which are known carcinogens [32]. Further, additives that give a “buttery” or “creamy” flavor can produce another potentially dangerous compound when heated called diacetyl [33–35]. Occupational exposure to diacetyl in food production facilities that produce microwave popcorn, cookies, cereal, chocolate, and coffee can cause bronchiolitis obliterans, commonly known as “popcorn lung” [36] [37–39]. This lung disease is characterized by fibroproliferative airway lesions and decrements in lung function [39, 40]. Thus, despite the public perception that flavor compounds are safe for human health, there is growing evidence that aerosolization could convert such seemingly safe compounds to chemicals that in fact pose potential human health hazards.

Not only could aerosolized e-liquid exposure be a health risk to an adult user but also to an unborn child. There are currently only a few studies that have tested the effects of ECIGs on developing embryos, but those that have indicate a potential hazard could exist. For example, zebrafish larvae exposed to aerosols produced by a tobacco flavored e-liquid result in heart defects [41]. In developing mice, the same flavored ECIG aerosol, results in the reduction of neurodevelopmental gene expression, especially growth factors, in the frontal cortex [42]. We also know that propylene glycol exposure alone can also cause a range of developmental defects, including smaller size, in zebrafish embryos [43].

In order to determine if the chemicals produced by ECIG aerosolization affects embryonic development, specifically craniofacial formation, we have turned to the developmental model *Xenopus laevis*. This species is amenable to screening studies since they are free living, large in size, develop rapidly, and can be obtained in great numbers [44] add more refs. Further, *Xenopus* is emerging as an effective model to understand craniofacial development [45–52]. The orofacial region is readily visible, unlike other model vertebrates where head flexure obscures easy viewing of the face, making evaluation of craniofacial development faster and easier [45]. Further, we have also developed a quantitative method to assess *X*. *laevis* orofacial morphology that can uncover subtle differences in orofacial shape and size [53–56].

In the present study we investigate whether chemicals produced by e-liquid aerosolization can adversely affect embryonic development, in particular craniofacial formation. Here, specific ECIG aerosol mixtures (e-cigAMs) that perturb orofacial morphology, cranial cartilage and muscle development as well as reduce cranial blood formation were identified. The same particular e-cigAMs also reduce the expression of vascular and cartilage differentiation factors and cell vitality in mammalian neural crest cells. The results emphasize the need for further testing of e-liquids, since they could cause serious craniofacial birth defects such as cleft palate.

## Materials and methods

### E-cigAM preparation

**Fig 1:**
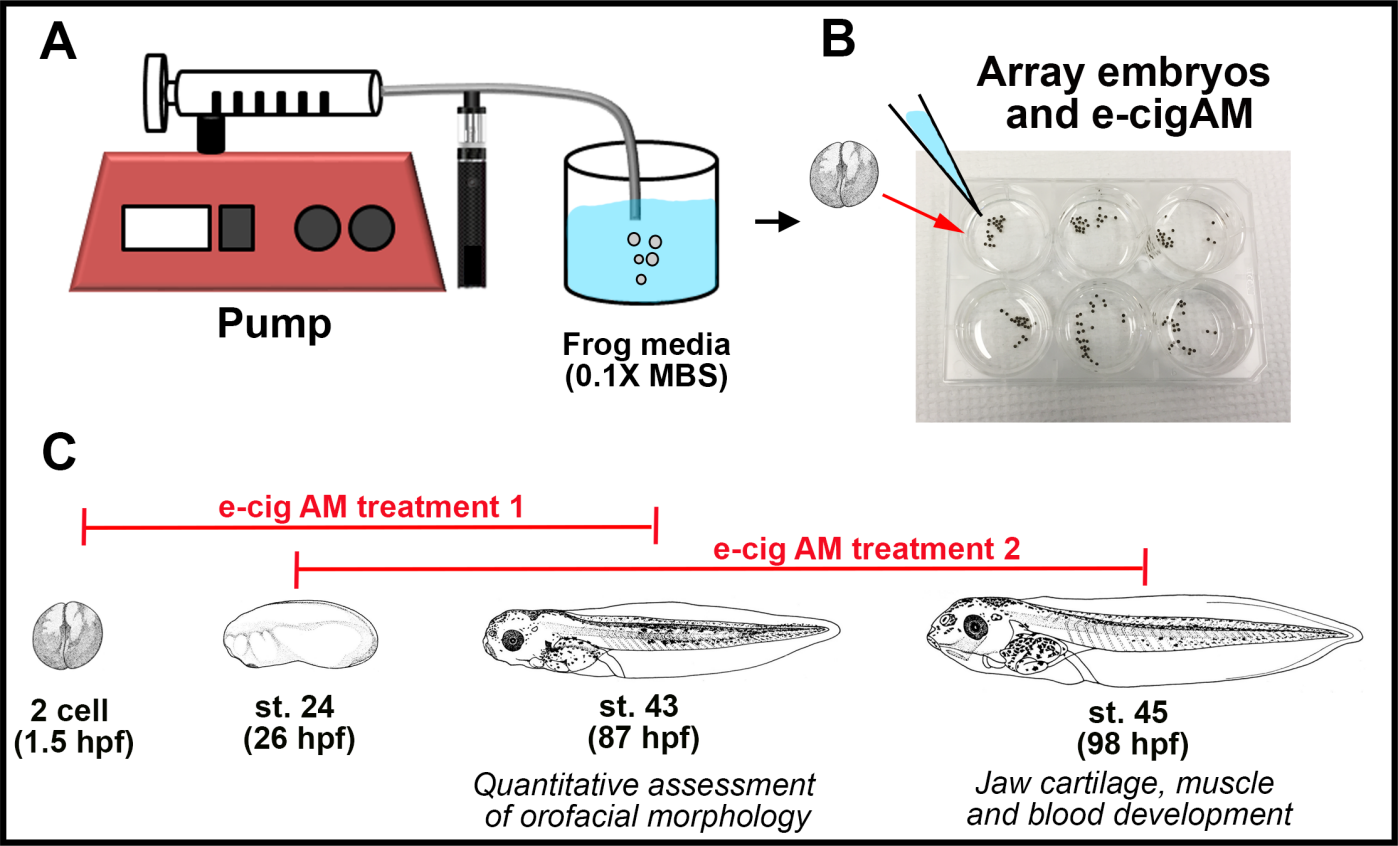
Experimental setup for e-cigAM collection. **(A)** Preparation of e-cigAM stock solution. ECIG vapor was aerated into 1 mL of 0.1X MBS by a 1.6 ohm atomizer attached to a syringe pump following a topography profile of: 3.5-second puffs (55 mL)with 30 second intervals between each puff for a series of 10. **(B)** Experimental set-up for *X*.*laevis* embryos. Embryos were arrayed in 6-well culture dishes in a 1:100 dilution of e-cigAM in 0.1X MBS. **(C)** E-cigAM exposure paradigm for qualitative and quantitative analysis. Embryos were exposed to e-cigAM from the 2-cell stage (1.5 hpf) to stage 43 (87 hpf).

Aerosolized e-liquid mixtures (E-cigAM) was prepared by attaching a 1.6 ohm atomizer (Aspire K2 Quick Starter Kit, 1.8 mL tank) into a syringe pump (NE-8000, New Era Pump Systems Inc., Farmingdale, NY) and aerating ECIG vapor into 1 mL of phosphate buffered saline (PBS) for cell studies, and 1 mL of 0.1X Modified Barth’s Saline Solution (MBS) for *in-vivo* studies (Fig 1A). E-cigAMs were generated using Lab Grade or a variety of flavored e-liquids (Table 1). The topography used to generate the e-cigAM was based on recent reports of topography of experienced users [57]. Briefly, a series of 10 3.5-second puffs (55 mL) with 30 second intervals between each puff was considered one “vaping session”. The e-cigAM solutions were then diluted in cell culture media or 0.1X Modified Barth’s Saline Solution (MBS) to achieve the desired experimental concentrations. E-cigAM was prepared the day before use and stored at -80° Celsius until needed.

**Table 1:** The e-cigAMs tested on *X*. *laevis* embryos. The nicotine concentration in mg/ml, ratio of propylene glycol to vegetable glycerin, and flavor additives are as reported on packaging.

ecigAM	Derived from E-liquids with:		
	Nicotine conc. (mg/ml)	PG/VG Ratio	Known Flavors
**A**	24	70/30	Red Tobacco
**B**	24	70/30	Dark chocolate, Milk Chocolate
**C**	18	30/70	Melon, Candy
**D**	24	70/30	Menthol
**E**	18	30/70	Strawberry, Almond, Caramel, Vanilla, Biscuit, Vienna cream
**F**	6	30/70	Cereal, Berries, Cream, Citrus

### *X*. *laevis* embryo e-cigAM exposures

Embryos were collected using standard procedures [58] approved by the VCU Institutional Animal Care and Use Committee (IACUC protocol number 5AD20261). Eggs were collected from two different females for each biological replicate. Experiments were set up in a laminar flow hood using semi-sterile conditions, in which autoclaved 0.1X Modified Barth’s Saline solution (MBS, pH 7.8), and UV-sterilized well dishes and transfer pipettes were used. In treatment paradigm 1, embryos were arrayed in 4 mL of 0.1X MBS in a 6-well culture dish with 40 μL (1:100 dilution) of e-cigAM stock solutions (Table 1). The media and e-cigAM was refreshed the next day. At stage 35 (50 hours post fertilization, hpf), embryos were transferred to 15 mm Petri dishes containing the same 1:100 dilution of e-cigAM stock solution in 0.1X MBS for a final volume of 15 mL. For craniofacial tissue analyses, e-cigAM exposures began at stage 22–24 (24–26 hpf) using the same procedures. All embryos were maintained at 23°C in an Echotherm incubator (Torrey Pines Scientific, cat #: IN30) throughout treatment until stage 43 or 45 for further analysis (88–98 hpf; Fig 1B and 1C).

### Quantifying orofacial shape and size

Once embryos reached stage 43 (88 hpf), they were fixed in 4% paraformaldehyde (PFA) overnight at 4°C. A sterile disposable No. 15 scalpel (VWR, cat. no: 82029–856) and Dumont No. 5 forceps (Fisher, cat. no: NC9404145) were used to make two cuts to isolate the head; first, at the posterior end of the gut and then second just behind the gills. Isolated heads were then mounted in small holes in a custom made clay-lined dish containing Phosphate Buffered Saline Solution with Tween (PBT) [53]. The faces were imaged using a Discovery.V8 stereoscope fitted with a GmbH digital camera (Zeiss). AxioVision40 V.4.8.1.0 software (Zeiss) and ImageJ (NIH) were used to perform all standard measurements. Such measurements included the: 1) *intercanthal distance*, which is the distance between the eyes, measured in Axiovision software, 2) *face height*, which is the distance between the top of the eyes and the top of the cement gland at the midline, measured in Axiovision software, 3) *mouth roundness*, which is the inverse aspect ratio of *(4area)/(major axis*^*2*^*)* applied to mouth outlines, measured in ImageJ, and 4) *dorsal mouth angle*, which is the angle created by drawing lines from the dorsal midline of the mouth to each labial commissure, measured in ImageJ. For all facial measurements, Student’s t-test assuming unequal variance between each e-cigAM group and controls was performed using VassarStats to determine statistical relationships. For bar graphs, data collected for the e-cigAM exposure groups were scaled so that the controls equaled 100 for graphical comparison. Error bars represent standard error calculated in Excel. Whisker plots were created using SigmaPlot software and whiskers represent minimum and maximum data points.

A total of 33 landmarks were placed on tadpole faces using the Pointpicker plugin of ImageJ (NIH) (S1 Fig). Landmarks 1–5 were placed on the midline of the mouth and the face between the top of the cement gland and the top of the eyes. Landmarks 6–17 outlined each eye. Landmarks 18–27 completed the outline of the midface region from the center of the eyes to the top of the cement gland, eliminating variation due to forebrain or cement gland size. Landmarks 22–33 outlined the mouth opening. Landmark coordinates were analyzed in MorphoJ 1.05f [59] via Procrustes superimposition to remove information concerning scale or size, position, and orientation. To study statistical relationships among all e-cigAM exposure groups and controls, a canonical variate analysis of Procrustes fit landmark data was performed and visualized as a bivariate plot. Discriminate function analysis was also performed to examine statistical relationships between each e-cigAM exposure group and its respective control. These were represented as transformation grids, in which the flat end of the vector represented average landmark position in control embryos, and the round end of the vector represented average landmark position in e-cigAM exposed embryos. Scale factor was set to 10.

### Analysis of blood distribution

Differentiated red blood cells were visualized using benzidine-peroxidase staining [60]. Benzidine forms a blue precipitate upon oxidation by the heme group of hemoglobin in the presence of hydrogen peroxide and thus serves as a histochemical stain specific for differentiated red blood cells. A 4X benzidine stock was made consisting of 2% glacial acetic acid and 0.4% benzidine (Sigma #B-3503) in distilled water. This solution was stored at 4°C in the dark. Embryos were immersed in 4 mL of 1X benzidine/acetic acid solution (diluted in distilled water) for 5 minutes. To initialize the color reaction, 40 μl of 30% hydrogen peroxide was added directly to the 1X benzidine/acetic acid solution containing the embryos. Cranial vasculature was clearly visible in control embryos after 5 minutes of the reaction and they were photographed immediately using a Discovery V8 stereoscope fitted with an Axiovision digital camera (Zeiss).

### Analysis of jaw cartilages and muscle

Cartilages were labeled with Alcian Blue using standard protocols with some modifications [61]. Briefly, stage 45 (98 hpf) tadpoles were fixed in Bouin’s fixative overnight at 4°C and then washed in 70% ethanol until clear of any yellow color. They were then immersed in Alcian Blue mixture (0.1mg/ml Alcian Blue in 1 part acetic acid: 4 parts ethanol) for 3–4 days at room temperature. Embryos were washed in 1% HCL in 70% ethanol for 1–2 days and cleared in 2% potassium hydroxide and glycerol.

Muscles were visualized using Phalloidin. Embryos at stage 45 (98 hpf) were fixed overnight in 4% PFA at 4°C followed by 3 washes (1 hour each) in PBT. Embryos were then labeled with Alexa Fluor 488 Phalloidin (1:100; Thermofisher, cat. no: A12379) for two days at 4°C and counterstained with Hoechst 33342 Solution to label nuclei (1:10,000; Thermo Fisher Scientific, cat. no: 62249). Embryos were imaged in 90% glycerol in PBT using a Nikon C2 confocal microscope. Z-stacks were created with a step size of 2 μm, for a total thickness between 200–400 μm. Using Elements software, Z-stacks were converted to maximum projection images for ventral views or volume rendered, rotated and 2D snapshot created for frontal views.

### Neural crest cell cultures

Murine cranial neural crest cells (O9-1, MilliporeSigma, St. Louis, MO) were seeded on hESC-qualified reduced growth factor basement membrane matrix (Geltrex®, Thermo Fisher Scientific, cat. No: A1569601) and culture in complete embryonic stem cell medium (MilliporeSigma, cat. No: ES-101-B). Cells were cultured to confluence and subsequently plated onto Geltrex® coated 24 well plates at a density of 20,000 cells/cm2. e-cigAM was diluted 1:100, 1:1000 and 1:10000 in culture media and this media was refreshed every 24 hours.

Cell health and viability was assessed with AlamarBlue® (Thermo Fisher Scientific, cat. No: 10-230-102). Briefly, 100 uL of AlamarBlue® was added to each well and incubated for 4 hours. After incubation, 100 uL of medium was removed and fluorescence intensity (560 nm EX/590 nm EM) was assessed. After measurement, fresh medium was added to each well with their respective e-cigAM treatments and dilutions.

Osteoblastic and chondrogenic differentiation was induced by culturing the neural crest cells for 12 days in osteoblastic and chondrogenic induction medium respectively (Lonza, Basel, Switzerland). Cells were plated in Geltrex® coated 24 well plates at 20,000 cells/cm2 in complete embryonic stem cells medium, osteogenic induction medium, and chondrogenic induction medium. Different dilutions of selected e-cigAM were added at plating and every 48 hours with fresh medium.

mRNA levels was assessed using quantitative RT-PCR. RNA was extracted from the cells using TRIzol® (Life Technologies) following manufacturer’s directions. RNA was reverse transcribed into cDNA using a High Capacity cDNA Kit (Life Technologies). Quantitative PCR was performed by combining established primers (available upon request) with PowerUp *SYBR*® Green Master Mix (ThermoFisher) and utilizing a CFX Connect Real-Time PCR Detection System (Biorad).

Data (six replicates per group) were analyzed by ANOVA and Student’s t-test with Bonferroni’s correction for multiple comparisons. P < 0.05 was considered to be significant.

## Results

### 1. "Lab Grade” e-cigAM containing only nicotine, propylene glycol, and vegetable glycerin have minor effects on orofacial development

**Fig 2:**
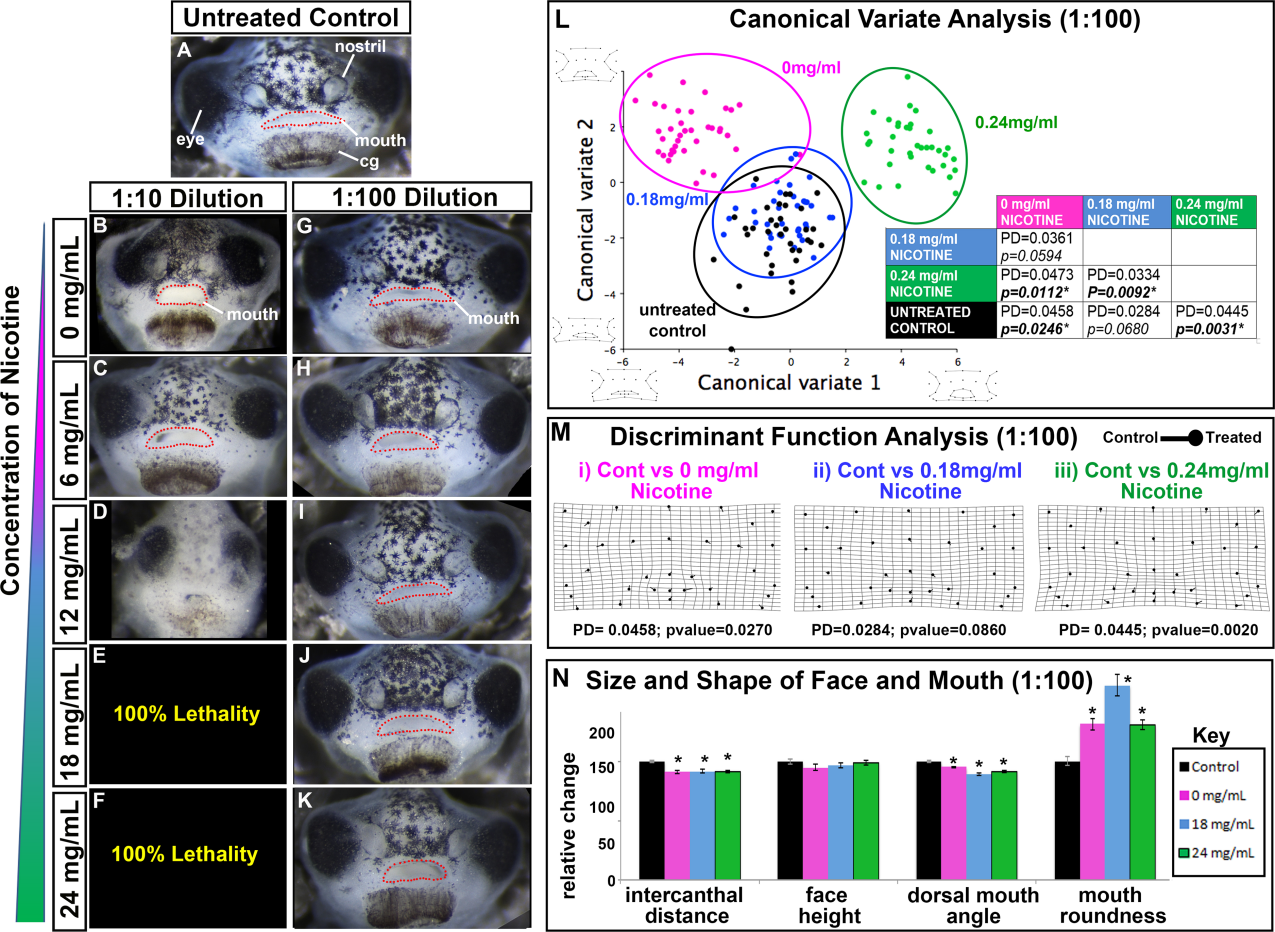
Effect of Lab Grade e-cigAMs on *X*. *laevis* orofacial development. **(A-K)** Representative frontal views of **(A)** untreated control, **(B-F)** 1:10, or **(G-K)** 1:100 dilutions of lab grade e-cigAMs containing (B,G) 0 mg/ml, (C,H) 6 mg/ml, (D,I’) 12 mg/ml, (E,J) 18 mg/ml, or (F,K) 24 mg/ml concentration of nicotine. Mouth outlined in red dots. **(L)** Canonical variateanalysis of controls and 1:100 dilution of Lab Grade e-cigAMs. Black = untreated controls, pink = Lab Grade e-cigAM with 0 mg/ml nicotine, blue = Lab Grade e-cigAM with 18 mg/ml nicotine, green = Lab Grade e-cigAM with 24 mg/ml. Table includes Procrustes distances (PD) and p-values. Significant p-values in bold with asterisks. Wireframe graphs represent shape changes associated with position on graph. Canonical variate 1 = 66.0% variance, Canonical variate 2 = 21.5% variance. **(M)** Discriminant function analysis of controls compared to Lab Grade e-cigAM with (i) 0 mg/ml, (ii)18 mg/ml, or (iii) 24 mg/ml concentration of nicotine. Flat end of vector is average landmark position of controls, round end is average landmark position of Lab Grade e-cigAMs. Procrustes distances (PD) and p-values below graphs. **(N)** Measurements of intercanthal distance, face height, dorsal mouth angle, and mouth roundness of controls (black), and 1:100 dilutions of e-cigAMs containing 0 mg/ml (pink), 18 mg/ml (blue), or 24 mg/ml (green) concentration of nicotine. Controls were set to 100 and exposure groups normalized to it. Student’s t-test assuming unequal variance was performed on non-normalized data. Error bars represent standard error. Asterisks indicate significant difference when compared to controls. Alpha value for all statistical analyses = 0.02. cg = cement gland.

Our first goal was to test aerosols generated from a “Lab Grade” e-liquid which contained only nicotine, propylene glycol (PG), and vegetable glycerin (VG) on the developing craniofacial region. We tested several dilutions of aerosolized e-liquid mixtures (e-cigAM) generated from the Lab Grade e-liquid with 0, 18, or 24 mg/mL concentrations of nicotine on embryos over an early developmental period (Fig 1C, treatment 1 paradigm). Unexposed controls were reared in 0.1X MBS and resembled wild type embryos (Fig 2A). Results revealed that a 1:10 dilution elicited very severe effects and lethality at high concentrations of nicotine (Fig 2B–2F). Similarly, a 1:50 dilution also caused lethality at high nicotine concentrations (not shown). At a 1:100 dilution we noted little lethality and minor defects in the shape of the mouth that was worse in embryos exposed to the Lab Grade e-cigAMs containing higher nicotine concentrations (Fig 2G–2K). Xenopus embryos treated with this dilution were also smaller consistent with reported effects of nicotine on human embryos (reviewed in [62, 63]). There were no noticeable effects on development when embryos were exposed to 1:1000 dilutions of Lab Grade e-cigAMs (not shown). These experiments indicated that a 1:100 dilution of e-cigAM could have a concentration of nicotine that is physiologically relevant to humans. Therefore, we used this dilution in all further experiments.

To assess changes in the shape of the orofacial region in embryos treated with 1:100 Lab Grade e-cigAM, we utilized geometric morphometrics. Thirty-three landmarks were chosen to best represent the shape of the orofacial region and incorporated features such as the position of the eyes and mouth (S1 Fig). Landmark data was then aligned by Procrustes fit to eliminate information about size and orientation, and analyzed using multivariate statistical analyses. We first performed a canonical variate analysis, which translates variance into components known as canonical variates and maximizes differences among groups by reducing the within-group variance [64]. When represented as a bivariate plot, statistical relationships among groups can be visualized such that groups that are highly different from each other will have a larger distance between them on the plot, large Procrustes distances, and small p-values. Embryos treated with Lab Grade e-cigAM containing 18 mg/mL of nicotine (blue circle) overlapped with the untreated controls (black circle) on the canonical variate analysis plot, suggesting a high degree of similarity in facial shape (Fig 2L). On the other hand, embryos treated with Lab Grade e-cigAM containing 0mg/mL (pink circle) or 24mg/mL (green circle) of nicotine were clearly discriminated from controls (Fig 2L).

In order to more specifically assess the differences in orofacial shape between embryos exposed to each Lab Grade e-cigAM compared to controls, we next performed a discriminant function analysis. In this pairwise analysis, results are represented as vectors diagrams, such that the flat end of the vector represents the average landmark position in control embryos, and the closed circle end represents the average landmark position in embryos exposed to each Lab Grade e-cigAM. The length of the vectors represents the magnitude of shape change, and warping of the transformation grid highlights regions of the most significant changes. The transformation grid of facial landmarks from embryos exposed to Lab Grade e-cigAM containing 0 mg/mL and 18mg/ml of nicotine revealed subtle shifts but were not deemed significant (p values above 0.01, Fig 2Mi, Mii). Embryos exposed to Lab Grade e-cigAM with 24 mg/mL of nicotine exhibited significant shifts in the position of mouth and ventral facial landmarks as well as warping of the transformation grid in the lower portion of the face (Fig 2Miii). Together, the Canonical variate and discriminant analyses suggest that Lab Grade e-cigAM containing 24 mg/ml of nicotine resulted in minor but significant changes in the shape of the orofacial region.

The morphometric analysis revealed some overall orofacial shape changes, especially surrounding the mouth, in the embryos treated with Lab grade e-cigAM with high nicotine. The shape of the mouth depends upon the amount palate and midface tissue and therefore we next performed additional measures of mouth shape. Normally, embryos have a broad oval shaped mouth while embryos with less midface tissue have rounder mouths. Mouth roundness was assessed using the inverse aspect ratio: *(4area)/(major axis*^*2*^*)*. In addition, we also measured the dorsal mouth angle; that is the angle which is created when lines are drawn from the dorsal midline of the mouth to each labial commissure (S1 Fig). An angle that is more acute can be indicative of a median cleft and/or a rounder mouth that can form when palate tissues are reduced. Embryos exposed to all Lab Grade e-cigAMs had rounder mouths (0mg/ml = 31.01%, p< 0.0001; 18mg/ml = 64.04%, p< 0.0001; 24 mg/ml = 31.43%, p< 0.0001, Fig 2N, S2 Fig), and more acute dorsal mouth angles (0mg/ml = 8.70%, p = 0.0002; 18mg/ml = 10.63%, p< 0.0001; 24 mg/ml = 4.71%, p< 0.0001; Fig 2N, S2 Fig). These results demonstrate that the faces of Lab Grade e-cigAM treated embryos do all have minor changes in the shape of the mouth, reflecting a possible reduction of midface tissues.

Morphometric and mouth shape analyses effectively revealed orofacial shape changes but these measures do not reveal changes in size. Therefore, the intercanthal distance (the distance between the eyes) and the facial height (distance from the top of the cement gland to the top of the eyes) were measured (S1 Fig). Embryos exposed to all Lab Grade e-cigAMs had slightly but statistically decreased intercanthal distances (0mg/ml = 8.47%, p< 0.0001; 18mg/ml = 8.55%, p< 0.0001; 24mg/ml = 9%, p< 0.0001; Fig 2N, S2 Fig), but no significant changes in the face height (p’s = 0.149; 0.241; 0.670 respectively; Fig 2N, S2 Fig). These results suggest that each of the Lab Grade e-cigAMs, even in the absence of nicotine, resulted in slightly narrower midfaces.

Taken together, these quantitative analyses support our qualitative observations that Lab Grade e-cigAMs induce some subtle defects in orofacial morphology, especially surrounding the mouth, that were more significant at the highest concentration of nicotine.

### 2. E-cig AMs containing flavors differentially affect craniofacial development

In addition to PG, VG, and nicotine, a variety of flavor additives are added to e-liquids. Therefore, we asked whether various e-cigAMs containing flavor additives could have differential effects on craniofacial development. E-cigAMs derived from six e-liquids were randomly chosen with different numbers or types of flavors, different or unknown PG/VG ratios and different levels of nicotine (summarized in Table 1). The goal of this section is not to determine which components of the e-liquids are toxic, but rather to identify whether some e-liquids could dramatically affect craniofacial development.

**Fig 3:**
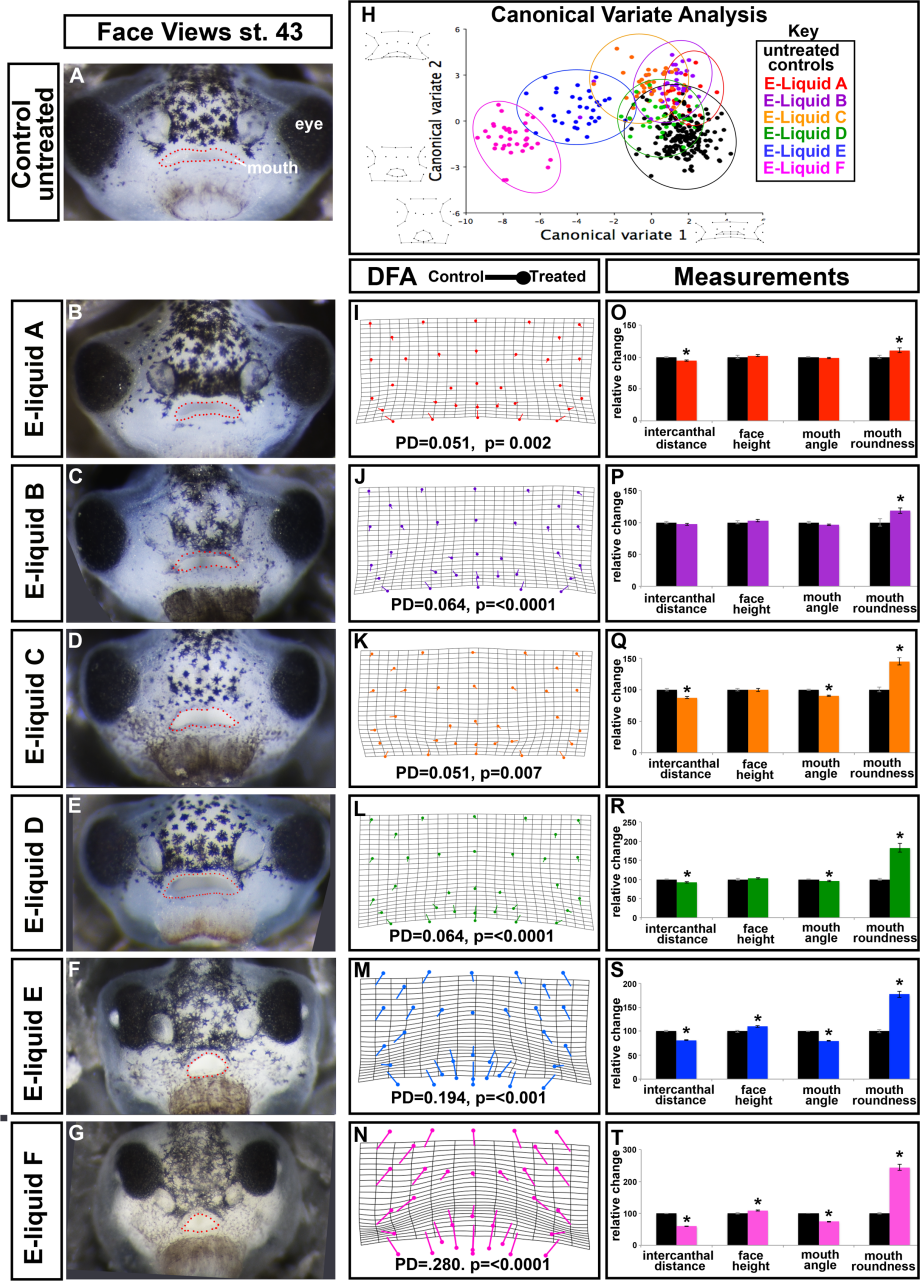
Effect of flavored e-cigAMs on *X*. *laevis* orofacial development. **(A-G)** Representative frontal views of (A) untreated controls, (B) embryos exposed to e-cigAM A, (C) embryos exposed to e-cigAM B, (D) embryos exposed to e-cigAM C, (E) embryos exposed to e-cigAM D, (F) embryos exposed to e-cigAM E, and (G) embryos exposed to e-cigAM F. Mouth outlined in red dots. **(H)** Canonical variate analysis of controls and e-cigAMs A-F. Black = untreated controls, red = e-cigAM A, purple = e-cigAM B, yellow = e-cigAM C, green = e-cigAM D, blue = e-cigAM E, pink = e-cigAM F. Wireframe graphs represent shape changes associated with position on graph. Canonical variate 1 = 68.8% variance, Canonical variate 2 = 15.8% variance. **(I-N)** Discriminant function analysis of controls compared to e-cigAMs A-F. Flat end of vector is average landmark position of controls, round end is average position of e-cigAMs. Procrustes distances (PD) and p-values below graphs. **(O-T)** Measurement of intercanthal distance, faces height, mouth angle, and mouth roundness of controls and e-cigAMs A-F. Controls were set to 100 and exposure groups normalized to it. Student’s t-test assuming unequal variance was performed on non-normalized data. Error bars represent standard error. Asterisks indicate significant difference when compared to controls. Alpha value for all statistical analyses = 0.02. cg = cement gland.

Embryos exposed to e-cigAM A-D exhibited only subtle orofacial defects, and largely resembled untreated controls and embryos treated with the Lab grade e-cigAMs (Fig 3A–3E). On the other hand, e-cigAMs E and F resulted in a much more distinct craniofacial phenotype, in which the lenses were protruding and the face appeared narrower, particularly the midface (Fig 3F and 3G). The mouth also appeared rounder in these embryos, with a median oral cleft in the “upper lip” region that extended into the roof of the oral cavity (Fig 3F and 3G, arrowheads) in 20% of embryos treated with e-cigAM E (n = 40; 2 replicates; Fig 3F) and 70% of embryos exposed to E-liquid F (n = 50; 3 biological replicates; Fig 3G).

While our qualitative observations identified two e-cigAMs that resulted in dramatic craniofacial defects, we next aimed to more thoroughly quantify these observations. First, to address the overall changes in the shape of the orofacial region, we utilized geometric morphometric analysis of e-cigAM exposed embryos and controls. Canonical variate analysis showed that control embryos (black circle) were not obviously discriminated from embryos treated with e-cigAMs A-D on the plot (Fig 3H) and these had small Procrustes distances (Table 2). On the other hand, embryos exposed to e-cigAMs E (blue circle) and F (pink circle) were more easily discriminated from the other treatment groups (Fig 3H). Further, these two groups were statistically different than their respective controls and had the largest Procrustes distances (Table 2).

**Table 2:** Procrustes distances and p-values derived from canonical variate analysis of controls compared to each e-cigAM exposure.

	Control	e-liquid A	e-liquid B	e-liquid C	e-liquid D
**e-liquid A**	0.0482 ***0*.*0005***				
**e-liquid B**	0.0441 ***0*.*0003***	0.0247 *0*.*4058*			
**e-liquid C**	0.0640 ***<0*.*0001***	0.0487 ***0*.*0088***	0.0392 *0*.*0437*		
**e-liquid D**	0.0639 ***<0*.*0001***	0.0423 ***0*.*0236***	0.0465 ***0*.*0123***	0.0332 *0*.*0528*	
**e-liquid E**	0.2061 ***<0*.*0001***	0.1779 ***<0*.*0001***	0.1757 ***<0*.*0001***	0.1480 ***<0*.*0001***	0.1428 ***<0*.*0001***
**e-liquid F**	0.2890 ***<0*.*0001***	0.2626 ***<0*.*0001***	0.2596 ***<0*.*0001***	0.2314 ***<0*.*0001***	0.2267 ***<0*.*0001***

Discriminant function analysis of embryos exposed to e-cigAMs A-D compared to their control counterparts generated minimal shifts in landmark positions and small Procrustes distances (Fig 3I–3L). This suggests that shape changes induced by these e-cigAMs were small compared to controls, consistent with the canonical variate analysis and qualitative observations. On the other hand, the discriminant function analysis of embryos exposed to e-cigAM E or F resulted in significant shifts in most landmark positions and a large Procrustes distances compared to the control embryos (Fig 3M and 3N). The landmark shifts and warping indicate an overall thinner face and triangular rather than oval mouth shape in these two groups (Fig 3M and 3N).

In summary, the canonical variate and discriminant function analyses indicate that embryos exposed to e-cigAMs E or F resulted in dramatic changes in overall face shape, consistent with the midface hypoplasia and median clefts observed. On the other hand, e-cigAMs A-D exposure lead to little or no overall changes in face shape.

To address changes in the size of the midface and more specifically assess changes in mouth shape, we measured intercanthal distance, facial height, dorsal mouth angle, and mouth roundness as described above in section 1. Embryos treated with e-cigAM A had a slightly smaller intercanthal distance (2%; p< 0.0001) and no significant change in the face height (p = 0.473), dorsal mouth angle (p = 0.149), or mouth roundness (p = 0.033; Fig 3O, S3 Fig). These results suggest only minor changes in the faces of these embryos. Embryos treated with e-cigAM B had no difference in the intercanthal distance (p = 0.265), face height (p = 0.319) dorsal mouth angle (p = 0.074), or mouth roundness (p = 0.016; Fig 3P, S3 Fig), suggesting they were also not much different from their respective controls. Exposure to e-cigAM C induced a 12% (p< 0.0001) reduction in intercanthal distance, but no significant change in facial height (p = 0.964; Fig 3Q, S3 Fig). The mouth had a dorsal mouth angle 9.5% more acute and was 44% more round and in e-cigAM C exposed embryos compared to controls (p< 0.0001; Fig 3Q, S3 Fig). Thus, while morphometrics revealed little difference in the shape of the face after exposure to this e-cigAM, these results show that the face is smaller with specific changes in the shape of the mouth (Compare Fig 3H, 3K and 3Q, S3 Fig). Embryos treated with e-cigAM D had a slightly smaller intercanthal distance (7%; p = 0.001; Fig 3R, S3 Fig) and no significant change in the face height (p = 0.319) or dorsal mouth angle (p = 0.012). The mouths of e-liquid D treated embryos had an 82% increase in roundness (p<0.0001; Fig 3R, S3 Fig). Here again, these results show that the face is smaller with specific changes in the shape of the mouth despite the fact that morphometrics revealed little difference in the overall shape of the face. E-cigAM E induced an 18% (p< 0.0001) reduction in intercanthal distance, and a 10% increase in facial height (p = 0.001; Fig 3S, S3 Fig). E-cigAM E exposure resulted in mouths that were 20% more acute (p< 0.0001) and 77% more round (Fig 3S, S3 Fig). These results are consistent with changes in facial shape identified by morphometric analysis. Embryos treated with e-cigAM F had a much smaller intercanthal distance (40%; p< 0.0001) and a 9% increase in the face height (p = 0.002; Fig 3T, S3 Fig). Dorsal mouth angle was 25% more acute in e-liquid F (p<0.0001) and 144% more round (p<0.0001) than controls (Fig 3T, S3 Fig). These results are also consistent with the shape changes in the face as identified by geometric morphometric analyses.

We also tested these e-cigAMs over a different time window (Fig 1, treatment 2) where embryos were exposed from stage 24–45 (26–97 hpf). By doing this we could determine if the e-cigAMs effects were mainly due to perturbations during early developmental events such as gastrulation and neural tube formation. When embryos were exposed to e-cigAM A-D during treatment 2, little or no change in orofacial morphology was observed (not shown). However, when embryos were exposed to either e-cigAM E or F over treatment 2 the same dramatic orofacial phenotype with median clefts was observed (not shown). These results indicate that the effects of these two e-cigAMs could be specific to later developmental events such as facial prominence growth, differentiation and local cellular movements during prominence fusion.

In summary, exposure to e-cigAMs E and F generated dramatic changes in facial dimensions and shape, while the other flavored e-cigAMs (A-D) had only minor or negligible effects on the orofacial size and shape.

### 3. E-cigAMs E and F result in orofacial defects even in the absence of nicotine but high nicotine can exacerbate craniofacial abnormalities in e-cigAM E

The e-cigAMs tested thus far all contained nicotine, however we noted varying effects. Therefore, we postulated that the primary cause of the orofacial malformations was not simply nicotine exposure. We hypothesized that the other components such as flavors, PG and VG could be major contributors to the orofacial defects. Therefore, we next asked whether orofacial malformations could occur when nicotine was absent from e-cigAMs. Further, we were also interested in whether nicotine could exacerbate the effects elicited by the other components. To test these questions, embryos exposed to e-cigAMs E and F with and without nicotine were compared.

**Fig 4:**
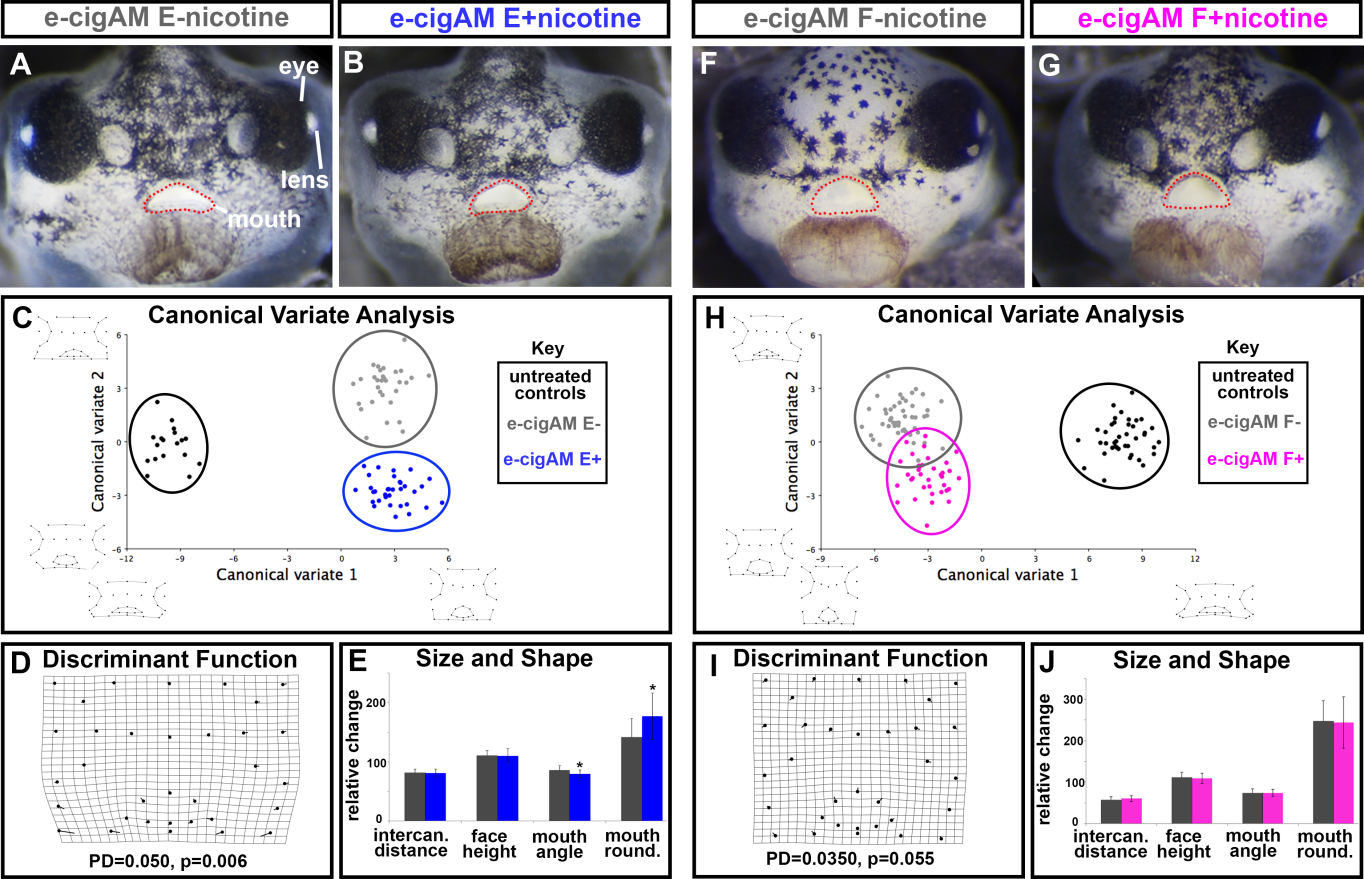
Effect of flavored e-cigAMs with and without nicotine on *X*. *laevis* orofacial development. **(A,B)** Representative frontal views of embryos exposed to (A) e-cigAM E- containing 0 mg/mL of nicotine or (B) e-cigAM E+ containing 18 mg/mL of nicotine. Mouth outlined in red dots. **(C)** Canonical variate analysis of controls (black), e-cig-AM E- (grey), and e-cigAM E+ (blue). Wireframe graphs represent shape changes associated with position on graph. Canonical variate 1 = 94.6% variance, Canonical variate 2 = 5.4% variance. **(D)** Discriminant Function analysis of e-cigAM E- compared to e-cigAM E+. Procrustes distances (PD) and p-values below graph. **(E)** Measurements of intercanthal distance, face height, mouth angle, and mouth roundness in e-cig-AM E- (grey), and e-cigAM E+ (blue). **(F,G)** Representative frontal views of embryos exposed to (F) e-cigAM F- containing 0 mg/mL nicotine or (G) e-cigAM F+ containing 6 mg/mL nicotine. (H) Canonical variate analysis of controls (black), e-cig-AM F- (grey), and e-cigAM F+ (pink). Wireframe graphs represent shape changes associated with position on graph. Canonical variate 1 = 79.8% variance, Canonical variate 2 = 20.2% variance. **(I)** Discriminant Function analysis of e-cigAM F- compared to e-cigAM F+. **(J)** Measurements of intercanthal distance, face height, mouth angle, and mouth roundness in e-cig-AM F- (grey), and e-cigAM F+ (pink). For bar graphs of size and shape measurements, controls were set to 100 and exposure groups normalized to it. Student’s t-test assuming unequal variance was performed on non-normalized data. Asterisks indicate significant difference when compared to controls. Alpha value = 0.02.

Embryos exposed to e-cigAM E with nicotine (e-cigAM E+) or without (e-cigAM E-) had orofacial defects, and neither group resembled controls (see representative control in Fig 3). Those embryos having a median cleft occurred at approximately the same frequencies (18% and 20% respectively, n = 40, 2 biological replicates for each experiment). However, defects in embryos treated with e-cigAM E+ appeared to to be slightly more severe than embryos treated with e-cigAM E-, and there was a slightly higher rate of lethality in this group (10%, compared to 7.5% in e-cigAM E-; Fig 4A and 4B).

Quantitative analyses comparing the orofacial shapes from embryos treated with e-cigAM E+ and e-cigAM E- confirmed our observations. The canonical variate plot indicates that the e-cigAM E+ (blue circle) and e-cigAM E- (grey circle) groups were discriminated from the controls (black circle) (Procrustes distances = 0.194 and 0.190 respectively; p’s< 0.0001; Fig 4C). These results indeed suggest that components in e-cigAM E cause orofacial defects even in the absence of nicotine. In addition, embryos treated with e-cigAM E+ (blue circle) and e-cigAM E- (grey circle) were discriminated from each other (Procrustes distance = 0.050; p = 0.012; Fig 4C), suggesting that nicotine exacerbates the orofacial shape changes caused by e-cigAM E (Fig 4C). A DFA comparing e-cigAM E+ and ecigAM E- indicated that overall the landmarks placements between these groups were significantly different (procrustes distance = 0.049, p = 0.006, Fig 4D). Notable landmark shifts were apparent in the ventral region of the face and around the mouth (Fig 4D). Consistently, there was a 7% decrease in mouth angle (p = 0.0003) and a 24% increase in mouth roundness (p< 0.0001) when embryos were exposed to the nicotine-containing e-cigAM E+ compared to e-cigAM E- (Fig 4E, S4 Fig). On the other hand, there was no statistical difference in intercanthal distance or facial height between embryos treated with nicotine containing e-cigAM E (p = 0.850, 0.864, respectively; Fig 4E,S4 Fig). Thus, while there is no difference in the size of the midface, the overall facial shape, especially around the mouth was different in e-cigAM E+ and ecigAM E- treated embryos. These results indicate that nicotine does exacerbate the craniofacial abnormalities.

Embryos exposed to e-cigAM F with nicotine (e-cigAM F+) and without nicotine (e-cigAM F-) had orofacial defects that appeared very similar, and did not resemble the controls (see representative control in Fig 3 and Fig 4F and 4G). In addition, 70% of embryos in both groups also exhibited median facial clefts (n = 50, 3 biological replicates; Fig 4F and 4G). In these two groups we could not discern any obvious difference; however, there was a slightly higher rate of lethality when exposed to e-cigAM F+ (14% compared to 4% in e-cigAM F-). A canonical variate plot indicates that the e-cigAM F+ (pink circle) and e-cigAM F- (grey circle) groups are discriminated from the controls (black circle) (procrustes distance = 0.2817, 0.311, respectively; p< 0.0001; Fig 4H). This suggests that even in the absence of nicotine, this e-cigAM F contains compounds that can cause orofacial defects. Embryos treated with e-cigAM F+ (pink circle) and e-cigAM F- (grey circle) overlapped and were not discriminated from each other (Procrustes distance = 0.034, p = 0.059; Fig 4H). A DFA comparison also revealed little shifting of landmark positions, accompanied by a low Procrustes distance, high p-value indicating no statistical difference between these groups (procrustes distance = 0.035, p = 0.055; Fig 4I). Additionally, there was no significant difference in intercanthal distance, face height, mouth angle, or mouth roundness of embryos treated with e-cigAM F with and without nicotine (p = 0.0658, 0.441, 0.830, 0.870, respectively; Fig 4J, S4 Fig). Taken together, these data suggests that the nicotine in this e-cigAM does not significantly contribute to orofacial defects (Fig 4). Note that in this e-liquid the reported nicotine concentration is low (6mg/ml) which may account for this result.

### 4. Orofacial shape in embryos exposed to ecigAM E differs from those exposed to e-cigAM F

**Fig 5:**
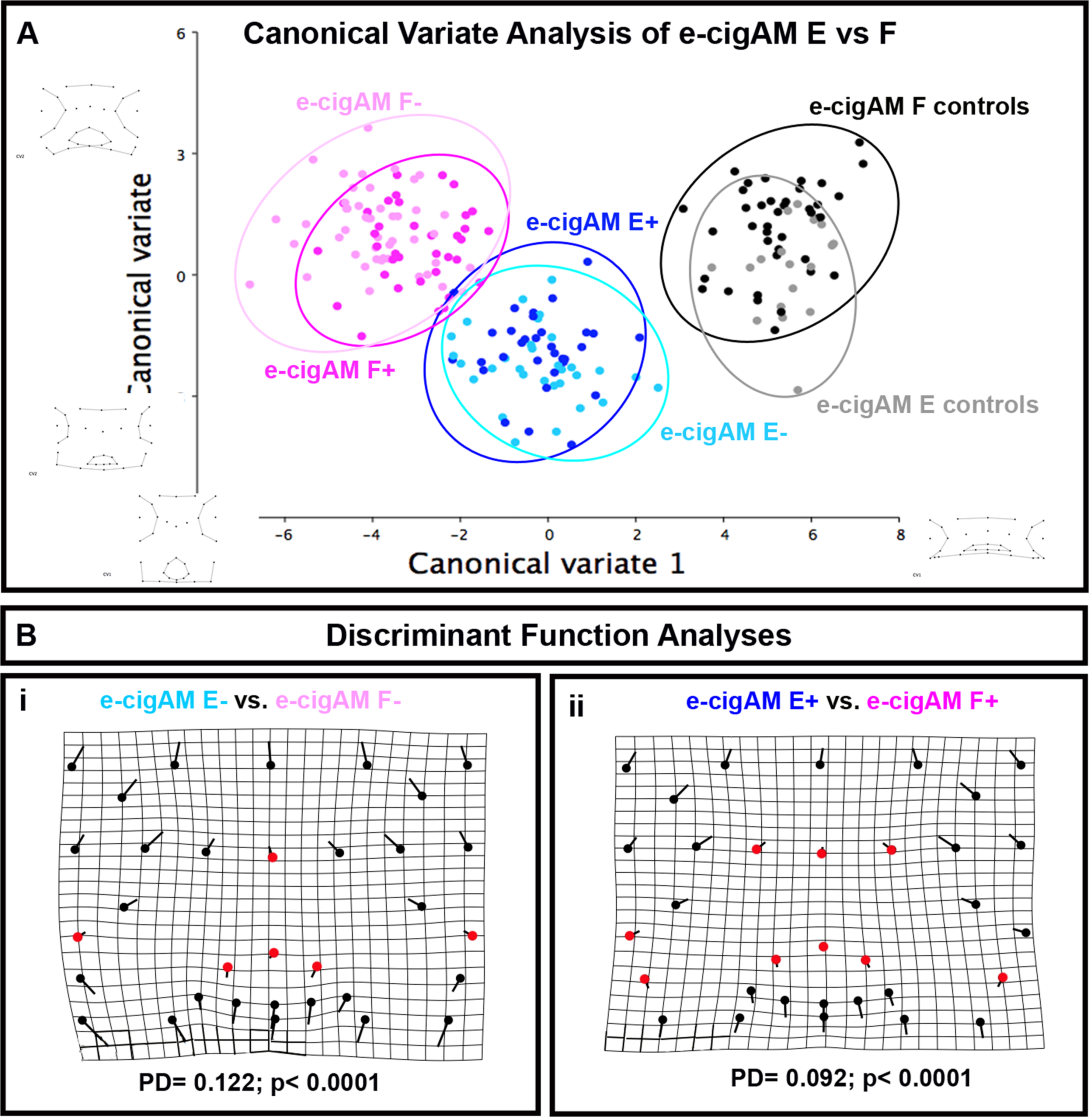
**A)** Canonical variate analysis. Comparison of e-cigAM E with nicotine (e-cigAM E+) or without nicotine (e-cigAM E-) and e-cigAM F with nicotine (e-cigAM F+) or without nicotine (e-cigAM F-). Dark blue = e-cigAM E+, light blue = e-cigAM E-, dark pink = e-cigAM F+, light pink = e-cigAM F-. Wireframe graphs represent shape changes associated with position on graph. Table contains Procrustes distance (PD) and p-values. Significant p-values are bolded and denoted with an asterisk. Canonical variate1 = 74.2% variance, canonical variate 2 = 11.0% variance. **B)** Discriminant Function Analysis of (i) e-cigAM E- compared to e-cigAM F-, (ii) e-cigAM E+ compared to e-cigAM F+. Flat end of vector is average landmark position of either e-cigAM E- or e-cigAM E+, round end is average position of e-cigAM F- or F+. Red dots are landmarks with little to no obvious change in position. Alpha value for all statistical analyses = 0.02.

Given the similarities in the orofacial phenotypes of embryos exposed to e-cigAM E or F, we also compared the shape changes in these groups. To do this a morphometric analyses of embryos with a phenotype were analyzed for overall changes in orofacial shape. First, a canonical variate analysis of embryos treated e-cigAM E and F with and without nicotine was performed. Results demonstrated that embryos exposed to e-cigAM E+ were for the most part discriminated from e-cigAM F+ on the bivariate plot (compare dark pink and dark blue circles). Some overlap existed due to one outlying embryo however these groups were statistically different (Fig 5A). Embryos exposed to e-cigAM E- were clearly discriminated from e-cigAM F- on the bivariate plot (compare light pink and light blue circles) and these groups were also significantly different (Fig 5A).

To more clearly visualize the specific differences in orofacial shape between e-cigAM E and F exposure groups a Discriminant function analyses was performed. When embryos from e-cigAM E- and e-cigAM F- were compared there were large shifts in landmarks throughout the face (black landmarks) and only minor shifts (red landmarks) at the midline of the face, dorsal mouth and at the periphery lateral to the mouth. We noted comparable results when embryos from e-cigAM E+ and e-cigAM F+ were compared. There were again minor shifts (red landmarks) at the midline of the face, dorsal mouth and at the periphery lateral to the mouth and larger shifts in landmarks throughout the face (black landmarks). These results suggest that both e-cigAM E and F without and without nicotine had similar effects on a very specific region of the midface accounting for the similarity in the qualitative phenotype but also caused very disparate effects on the rest of the embryonic face.

### 5. Exposure to either ecigAM E or F causes defects in cranial cartilage, muscle and blood distribution

Thus far, our analysis indicates that exposure to e-cigAM E and F cause quite dramatic changes in craniofacial morphology. We next wondered what specific tissues and structures might be perturbed that could account for such morphological changes. To determine this, we examined the major tissues comprising the jaw, namely muscle and cartilage as well as the blood supply to the face. These tissues are best examined at stage 45 (98hpf) when they are well formed. However, our treatment 1 paradigm resulted in variable but significant embryonic death by stage 45. Therefore, in this analysis we exposed embryos to e-cigAM E and F during a specific timeframe during which the orofacial region is developing (24–98 hpf, treatment 2 paradigm) to 1) ensure adequate numbers for analysis and 2) to more specifically test the effects of these e-cigAMs on orofacial development.

#### 5A) e-cigAM E or F exposure causes defects in cranial cartilages

**Fig 6:**
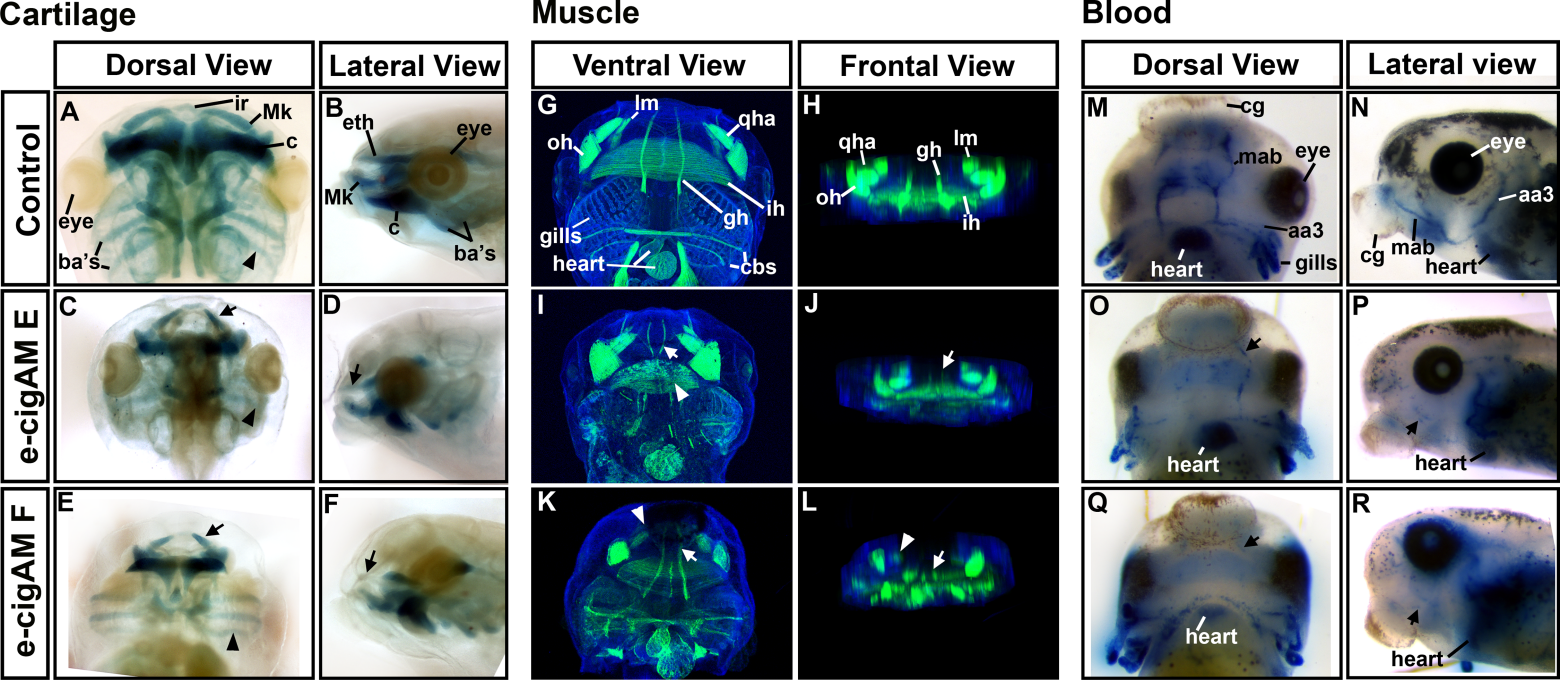
e-cigAMs E and F affect jaw cartilage, muscle and cranial blood distribution. **(A-F)** Cartilage labeling with Alcian Blue in representative embryos showing frontal views and lateral views in controls, e-cigAM E and F treated. Arrowheads are at right angles to the branchial cartilages. Arrows indicate the more obtuse angle of the infrarostral cartilages. **(G-L)** F-actin labeling of muscle (green) and Hoescht’s nuclear stain (blue) in representative embryos showing ventral views and frontal views in controls, e-cigAM E and F treated. Arrowheads indicate defects noted in the ih in I, and the lm in K and L. Arrows indicate defects in the gh in I J, K and L. **M-R)** Blood cell labeling with benzidine-peroxidase in blue of representative embryos showing frontal views and lateral views in controls, e-cigAM E and F treated. Arrows indicate deficicy in the mab in P and R. **Abbreviations:** ba’s = branchial cartilages, ch = cerathoyal, Mk = Meckel’s, ir = infrarostral, eth = ethmoid, qha = quadratohyoangularis, oh = orbitohyoideus, lm = levator mandibulae, ih = interhyoideus, gh = geniohyoideus, cbs = constrictors branchialium, mab = musculoabdominal vein, aa3 = third aortic arch, cg = cement gland.

The branchial and jaw cartilages that together form the cranial cartilage provide important structural support and allow for a functional mouth. At stage 45 (98 hpf), the anterior most infrarostral (ir) cartilage articulates with the paired Meckel’s (mk) cartilages on the upper jaw (Fig 6A and 6B). The ethmoid plate (eth) that supports the palate is present dorsal to the upper jaw structures (Fig 6A and 6B). At this time, the lower jaw is characterized by a separation of the paired cerathoyal (ch) cartilages by the basihyal (bh) cartilage at the midline. Finally, three pairs of branchial cartilages (ba) are present at this time (Fig 6A and 6B).

Embryos treated with either e-cigAM E or F, shared similar cartilage defects. For example, there was a reduction in the cranial cartilages (Fig 6A–6D, n = 20, 2 biological replicates) and the paired Meckel’s cartilages articulated with the infrarostral at a more acute angle in embryos treated with e-cigAM E or F when compared to their respective controls (Fig 6C and 6E, arrows). In addition, the branchial cartilages were positioned more perpendicular to the midline and this was more dramatic in embryos treated with e-cigAM E (Fig 6C and 6E, arrowheads are directed perpendicular to cartilage to show difference). Lateral views also indicated that the ethmoid plate is reduced in embryos exposed to e-cigAM E, and missing in embryos exposed to e-cigAM F (Fig 6D and 6F, arrows).

In summary, exposure to e-cigAMs E or F results in some similar abnormalities of the cranial musculature as well as some distinct differences specific to each e-cigAM.

#### 5B) e-cigAM E or F exposure causes defects in cranial muscle

Since the cranial cartilages were abnormal in embryos exposed to e-cigAMs E and F, we hypothesized that the muscles in the heads of these embryos might also be malformed. At stage 45 (98 hpf), the cranial muscles are composed of paired quadratohyoangularis (qha) and orbitohyoideus (oh) in the lower jaw and levator mandibulae (lm) in the upper jaw and the interhyoideus (ih) muscle extends laterally between these muscles. The paired geniohyoideus (gh) muscles are anchored anteriorly and extend over the interhyoideus (ih) to insert into the gill structures. Finally, the constrictors branchialium II,III, and IV (cbs) extend across the posterior part of the gills (Fig 6G and 6H) [65].

Embryos exposed to e-cigAMs E or F shared some similar defects, including the overall appearance of smaller muscle groups and changes in the spacing and arrangement of muscles (Fig 6G–6L). For example, in ventral views of the head we noted narrower geniohyoideus (gh) muscles that run anteroposteriorly at the midline (Fig 6I and 6K, arrows) that was especially apparent in embryos treated with e-cigAM E (Fig 6I). Exposure to this e-cigAM also resulted in disorganized interhyoideus (ih) muscle banding (Fig 6I, arrowhead) and missing constrictors branchialium (cbs; Fig 6I). Embryos exposed to e-cigAM F had smaller quadratohyoangularis (qha), orbitohyoideus (oh; Fig 6K), and levator mandibulae (lm) muscles (Fig 6K, arrowhead).

To more easily assess the muscle morphology after e-cigAM treatments, three dimensional images were created which could be rotated to view the muscles from different angles (see snapshots of the frontal views Fig 6H, 6J and 6L) From such images we noted that the geniohyoidus (gh) muscles in embryos treated with either e-cigAM E or F appeared to be in the correct location, but were much thinner than in the controls (Fig 6J and 6L arrows). In addition, it was clear that exposure to e-cigAM F resulted in severely reduced or missing levator mandibulae (lm) muscles (Fig 6L).

In summary, exposure to e-cigAMs E or F can cause similar abnormalities of the cranial musculature as well as differences specific to each e-cigAM.

#### 5C) e-cigAM E or F exposure causes a reduction in cranial blood cells

The blood supply carries oxygen throughout the body with the initiation of the heartbeat at stages 33–35 (44–46 hpf) [66, 67]. To determine if hemoglobin containing red blood cells reached the jaw after e-cigAM E and F exposures, we utilized benzidine-peroxidase labeling [68]. At stage 45 (98 hpf), red blood cell labeling was typically observed in high abundance in the heart and in regions consistent with the location of the aortic arches and the gills (Fig 6M and 6N). Erythrocytes were also present in two bilaterally symmetrical bands and more anteriorly around the mouth consistent with the location of the musculoabdominal vein (mab; Fig 6M and 6N). However, in embryos exposed to e-cigAMs E or F, blood cell labeling was decreased in the head in 100% of embryos (n = 20; 2 replicates; Fig 6M–6R). For example, in both groups of treated embryos, there was a clear reduction in the amount of blood cells associated with the third aortic arch and the musculoabdominal vein compared to controls (Fig 6O–6R, arrows). The blood cells that could be visualized in e-cigAM exposed embryos were observed in the same pattern as controls and did not appear diffuse (Fig 6O–6R). It could be argued that the lack of blood in the anterior region of the head is due to impaired heart function. However, blood was observed throughout the trunk and tail of these embryos in a similar manner to controls and we observed a heart beat in the e-cigAM E and F treated embryos (not shown).

In summary, our data reveals that e-cigAM E or F exposure affects the development of multiple orofacial tissues which could in turn accompany or cause the morphological abnormalities that we observed.

### 6. E-cigAM E or F result in misexpression of cartilage and vascular genes in mammalian neural crest cells

**Fig 7:**
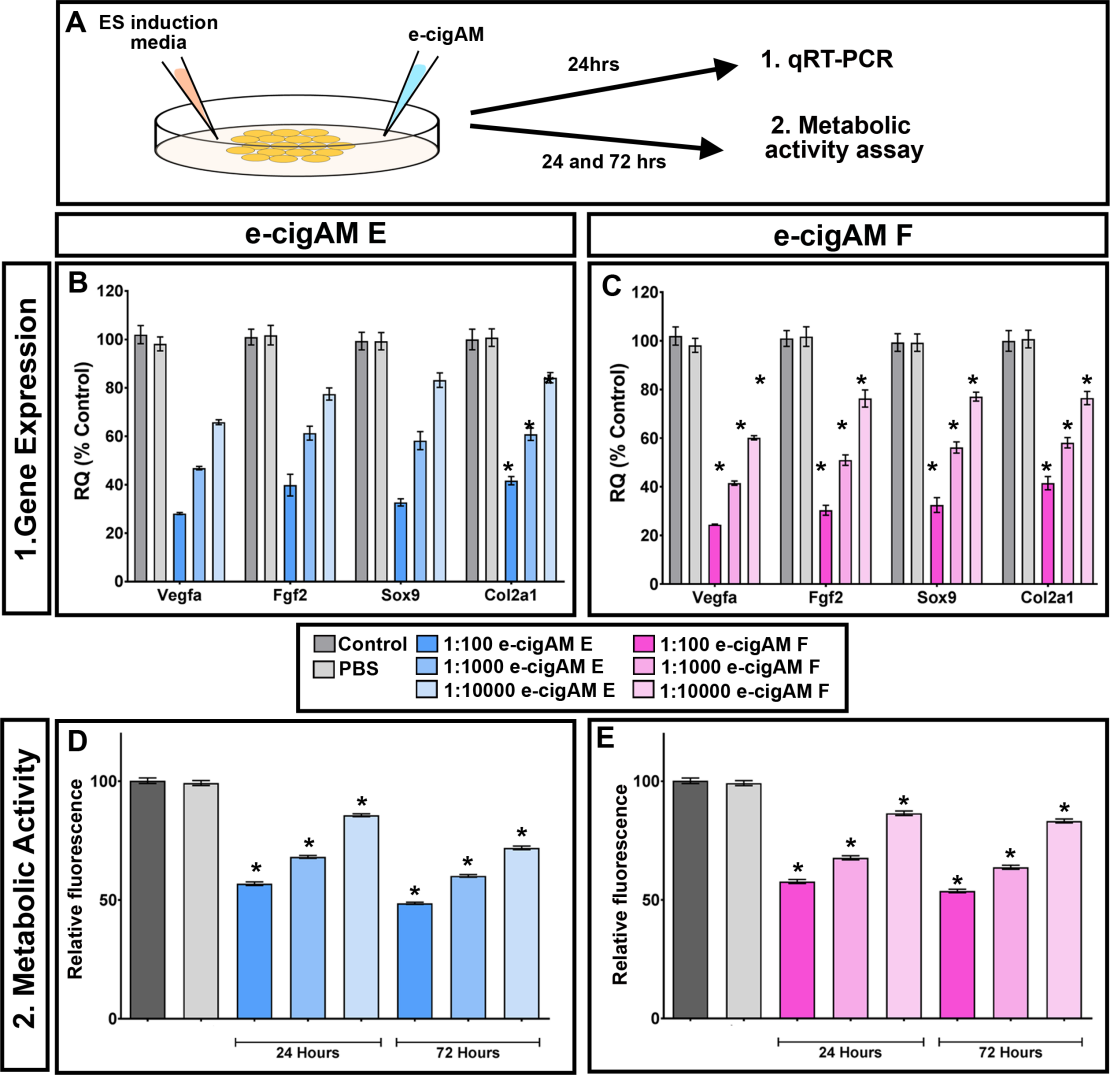
e-cigAM pertubs gene expression and cell health in murine neural crest cells. **A)** Experimental design. **B,C)** qRT-PCR showing normalized expression levels of vegf, fgf2, sox9 and Col2a1 in cells treated with e-cigAM E (B) and e-cigAM F(C). D,E) Alamar Blue assay showing relative fluorescence after 24 and 72 hours e-cigAM E (B) and e-cigAM F (C) treatment. Asterisks indicate significance.

Our results have demonstrated that embryos exposed to e-cigAM E or F had malformed cranial cartilage and a reduction in orofacial blood cells. To determine if these e-cigAMs could also affect mammalian systems in a similar way, their effects were tested in a murine neural crest cell line (O9-1). To do accomplish this, the expression levels of genes known to be critical to vascular and cartilage differentiation were assessed. Cells were stimulated to differentiate into osteoblast and chondrocytes with osteogenic and chondrogenic differentiation medium, and then exposed to e-cigAM E or F with nicotine at various dilutions for 12 days. Controls consisted of cells cultured on complete embryonic stem cell media as control and cells treated with PBS alone. Following e-cigAM exposure, murine neural crest cells were harvested, the RNA was extracted and qRT-PCR was performed (Fig 7A). Results revealed that the levels of the vascular differentiation marker, *vegf*, was statistically decreased in a dose dependent manner when exposed to both e-cigAM E or F (p value = < 0.05, 6 biological replicates, Fig 7B and 7C). Similarly, cartilage differentiation markers *fgf2*, *sox9* and *col2a1* were also statistically decreased when exposed to either e-cigAM E or F (p values = < 0.05, 6 biological replicates, Fig 7B and 7C). These results indeed suggest that cartilage and vascular differentiation may be perturbed in these neural crest cells.

We next wondered whether e-cigAMs could affect more generally neural crest cell health utilizing an Alamar Blue assay. In healthy metabolizing cells, a reducing environment is maintained where the Alamar Blue reagent, resazurin, is reduced to a fluorescent resorufin that can then be measured. A decrease in relative fluorescence would therefore indicate unhealthy, metabolocally less active cells. Cells were treated as described above where ES cell media was added along with e-cigAM E or F and fluorescence measured (Fig 7A). Results revealed that both e-cigAM E and F resulted in a decrease in metabolic activity in a dose dependent manner (p values = < 0.05, 6 biological replicates; Fig D,E).

Together, this data demonstrates that e-cigAM E or F could affect differentiation and viability of mammalian neural crest cells.

## Discussion

ECIG use has become a popular trend and it is believed by much of the general public to be a safer alternative to smoking [69–71]. Further, many women who vape during pregnancy do not believe it will harm the developing embryo and is not equatable to smoking cigarettes [7]. However, there is very little scientific data to support or dispel these ideas. Our work is the first to show that indeed ECIGs could perturb embryonic development, specifically craniofacial formation. Further, these effects seem to be translatable to mammalian systems. Thus, the data presented here emphasizes the need for more research focused on testing these popular products.

There are nearly 500 brands of e-liquids, and over 7,000 flavors [72] available from online providers and locally owned shops. It is this wide selection of flavors and brands that contributes to the appeal for ECIG consumers [2, 73]. While the number of options and flavors continues to increase, the impacts of heating the multitude of PG, VG and flavor combinations lags behind. Here, we have only scratched the surface in testing e-liquids; however, we have shown that 6 e-liquids can have differential effects on craniofacial development. Specifically, the aerosolized product of two of these e-liquids caused severe craniofacial abnormalities including a median cleft, whereas others resulted in only minor effects. Such disparate effect of different e-liquids has also been demonstrated in other systems. For example, four e-liquids consistently induced higher rates of cytotoxicity than other e-liquids in lung epithelial cells [74]. These four e-liquids had flavor components called Banana Pudding, Cinnamon, Menthol and Kola. Similarly, tests of a wide range of e-liquids in various types of cultured cells, including human embryonic stem cells, also revealed cytotoxic differences [75]. In this study the most highly cytotoxic e-liquids varied considerably and contained flavors such as berries, butterscotch and cinnamon. Further, they showed that such e-liquids do not affect all cell types in the same way. Our results showed that two e-liquids that caused severe craniofacial defects in embryos share common flavor additives, berries/strawberry and cream. In addition, we demonstrated that these two e-liquids had some subtle yet different effects on the shape of the face and the development of the cranial muscle and cartilage. Thus, we contribute to a growing body of data establishing that different chemicals produced in the aerosolization of e-liquids can have differential effects and these effects can vary across cell types. This is especially important when considering the embryo, where cells are continuously changing during the complex process of development. This accentuates the impossible need for testing e-liquids case by case and in whole animal systems. To complicate this further, not only are studies necessary to uncover the single most dangerous e-liquid components but also whether there is a potential for combinatorial effects.

Our goal in this study was to determine if e-cigAMs could affect craniofacial development and which tissues they might specifically affect. We determined that embryos exposed to two e-cigAMs (E and F) had dramatic craniofacial defects including eye abnormalities, midface hypoplasia, and a median cleft. These defects were accompanied by reductions in cranial cartilage and muscle, as well as a deficiency in the amount of blood cells in the facial tissue. While these craniofacial tissue types each have very different developmental origins they are each intimately tied to the development of the cranial neural crest [76, 77]. This developmental link would ensure that the skeletal, muscular and vascular jaw elements develop in concert, which in turn is critical for proper functionality [77, 78]. Certainly, we also determined that mammalian neural crest cell differentiation and metabolic activity were depressed when exposed to the same e-cigAMs that dramatically affect the embryos. Therefore our future work is directed toward determining how chemicals produced in the aerosolization of e-liquids affects developmental processes and whether neural crest specifically could be most sensitive.

In this study we hypothesize that the effects of the ECIG aerosols on Xenopus development are translatable to human embryos. Specifically, we believe that when a pregnant women vapes, the fetus would be exposed to a similar concentration of chemicals as the Xenopus embryos treated with e-cigAMs. Our first set of experiments, utilizing the “Lab Grade” e-cigAM containing only nicotine and carrier compounds, were utilized to determine relevant levels of nicotine. Most studies have demonstrated that human embryos exposed to nicotine alone have low birth weight and minor morphological defects [62, 63]. Consistently, we determined that a 1:100 dilution of “Lab Grade” e-cigAM resulted in smaller embryos with only minor craniofacial defects. These results suggest, based on the effects of nicotine, that Xenopus embryos treated with diluted e-cigAM could experience the same level of ECIG exposure as a human embryo. Future experiments in our labs will measure nicotine and other chemicals in the e-cigAMs as well as the amount of nicotine in Xenopus embryos to better establish human translational relevance.

## Conclusion

Our study is the first to show that exposure to ECIGs can have adverse effects on craniofacial development. This is of particular importance, since ECIGs are generally perceived by the public as “safe” for recreational use and only associated with mild effects when used during pregnancy. The work we have done here contradicts this perception and has the potential to inform public policy regarding the distribution and regulation of the ECIG market.

## Supporting information

S1 Fig Landmark and measurements performed in the study. (A) Landmarks (red dots) numbered in order of placement. (B) Measurements included face height (fh, green), intercanthal distance (id, red), dorsal mouth angle (dma, purple) and mouth roundness (mr, yellow dots outlining the mouth).
(TIF)

S2 Fig Whisker plots of the face measurements from embryos treated with “Lab Grade” e-cigAM. Whisker plots are shown for intercanthal distance, face height, dorsal mouth angle and mouth roundness. The median is represented by the line through the box, the top whisker represents the maximum value and the bottom whisker represents the minimum value. Asterisks represent statistically significant differences.
(TIF)

S3 Fig Whisker plots of face measurements from embryos treated with flavored e-cigAM. Whisker plots are shown for intercanthal distance, face height, dorsal mouth angle and mouth roundness for each e-cigAM A-F. The median is represented by the line through the box, the top whisker represents the maximum value and the bottom whisker represents the minimum value. Asterisks represent statistically significant differences.
(TIF)

S4 Fig Whisker plots of face measurements from embryos treated with e-cigAM E and F. Whisker plots are shown for intercanthal distance, face height, dorsal mouth angle and mouth roundness in the comparison of e-cigAM E and F with and without nicotine. The median is represented by the line through the box, the top whisker represents the maximum value and the bottom whisker represents the minimum value. Asterisks represent statistically significant differences.
(TIF)
