# Supplementary figures and images for: E-cigarette aerosol exposure can cause craniofacial defects in Xenopus laevis embryos and mammalian neural crest cells
Source: PLoS One. 2017 Sep 28;12(9):e0185729. doi: 10.1371/journal.pone.0185729 (PMC5619826; doi:10.1371/journal.pone.0185729)

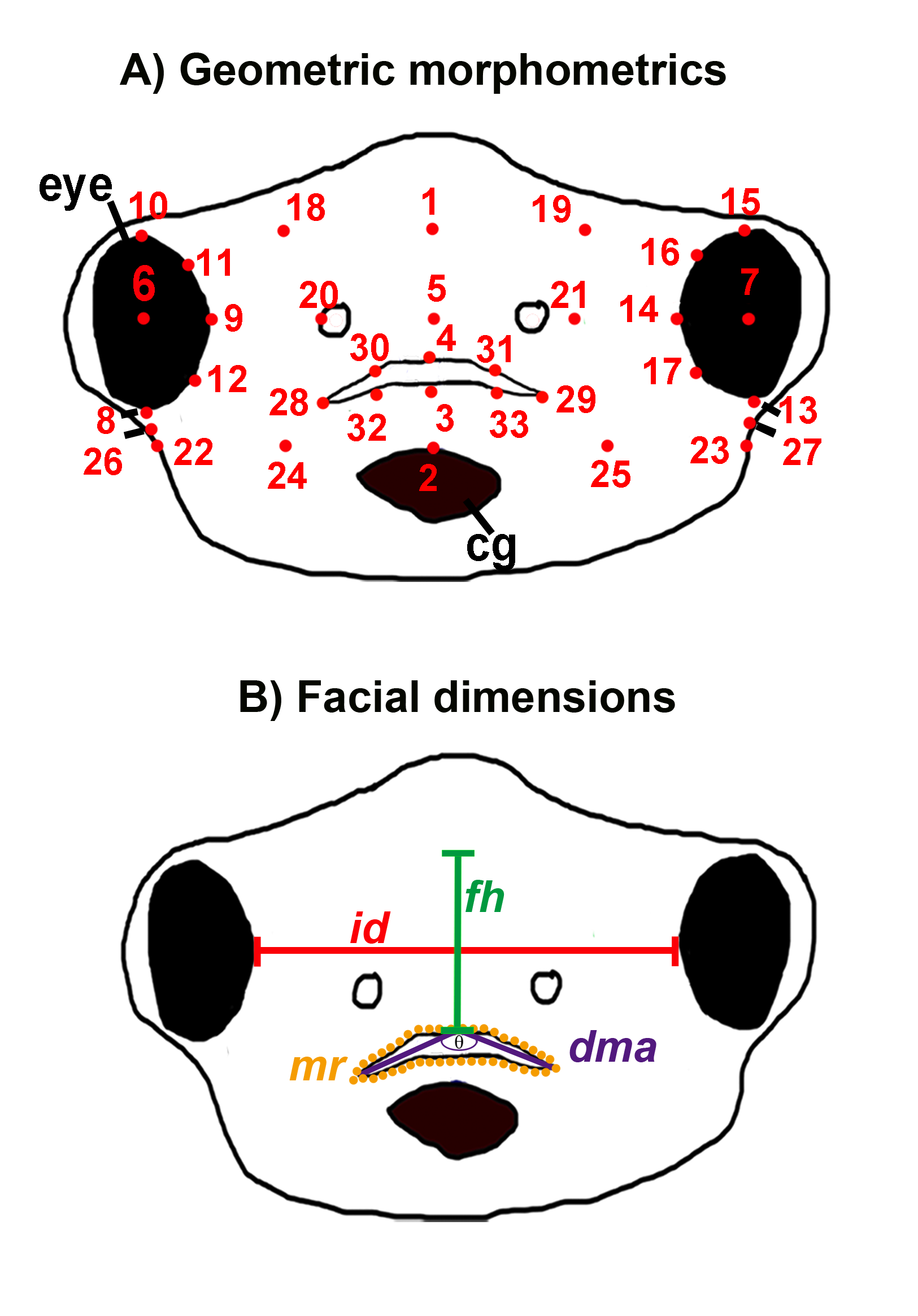

Supplement: S1 Fig — (A) Landmarks (red dots) numbered in order of placement. (B) Measurements included face height (fh, green), intercanthal distance (id, red), dorsal mouth angle (dma, purple) and mouth roundness (mr, yellow dots outlining the mouth). (TIF) [file pone.0185729.s001.tif]

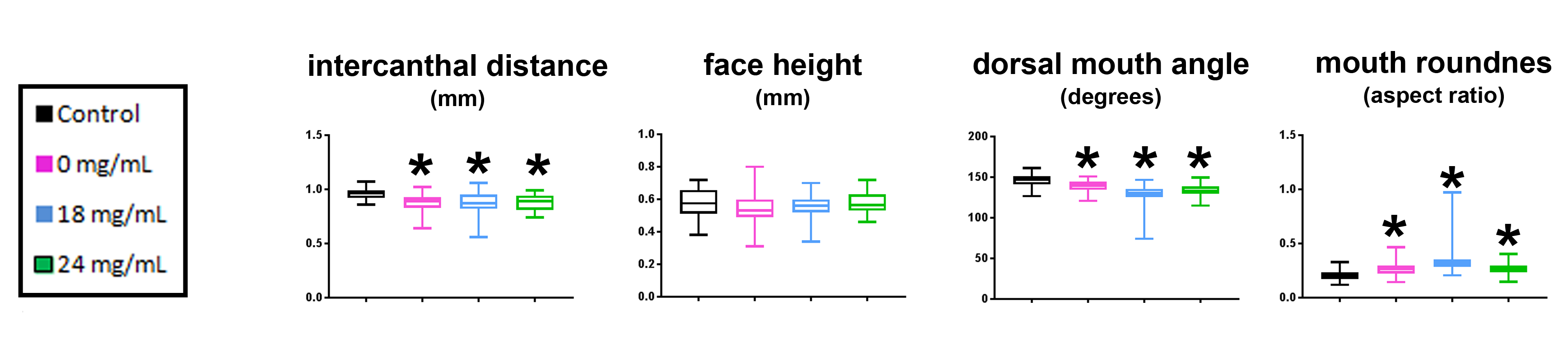

Supplement: S2 Fig — Whisker plots are shown for intercanthal distance, face height, dorsal mouth angle and mouth roundness. The median is represented by the line through the box, the top whisker represents the maximum value and the bottom whisker represents the minimum value. Asterisks represent statistically significant differences. (TIF) [file pone.0185729.s002.tif]

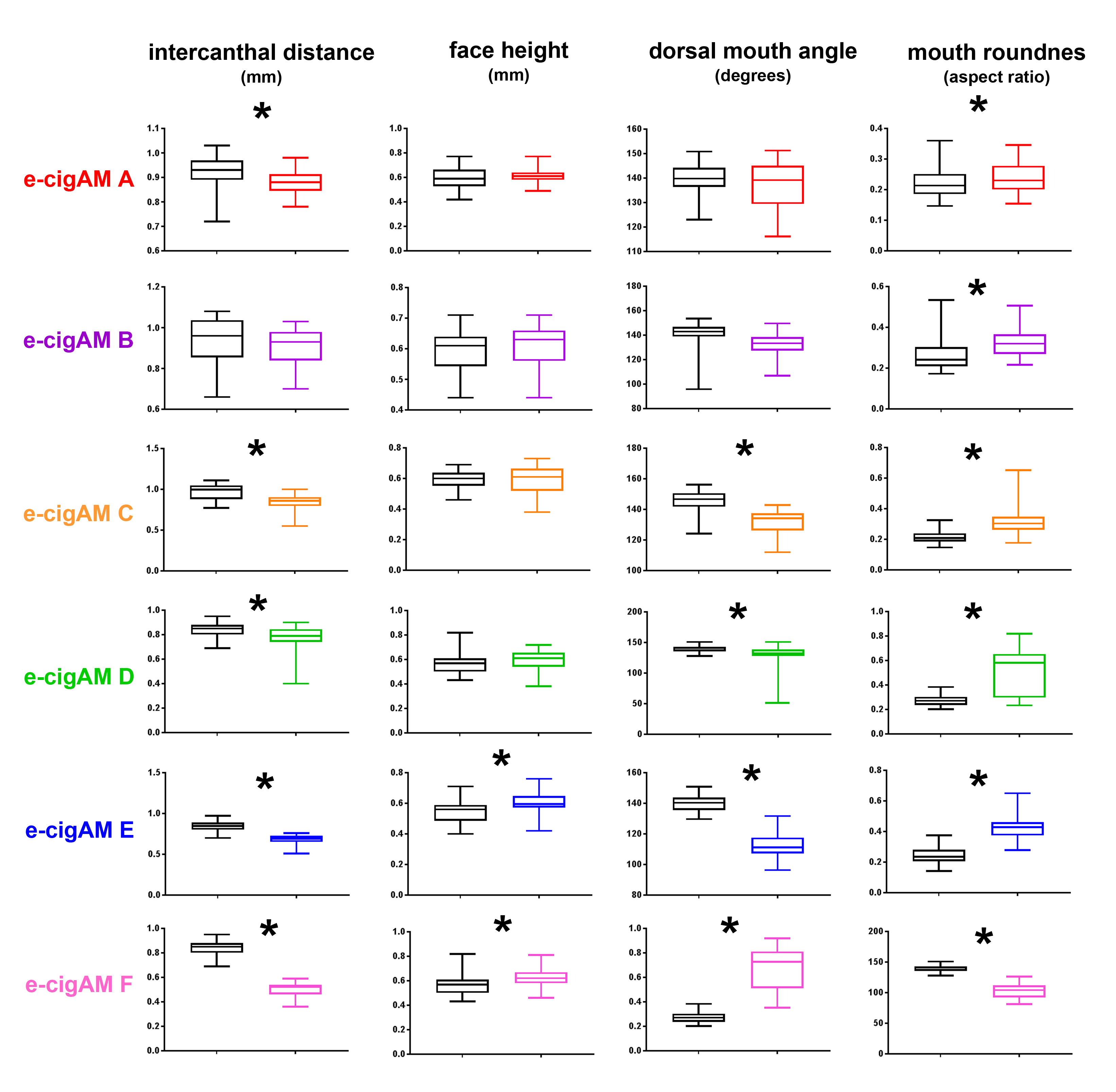

Supplement: S3 Fig — Whisker plots are shown for intercanthal distance, face height, dorsal mouth angle and mouth roundness for each e-cigAM A-F. The median is represented by the line through the box, the top whisker represents the maximum value and the bottom whisker represents the minimum value. Asterisks represent statistically significant differences. (TIF) [file pone.0185729.s003.tif]

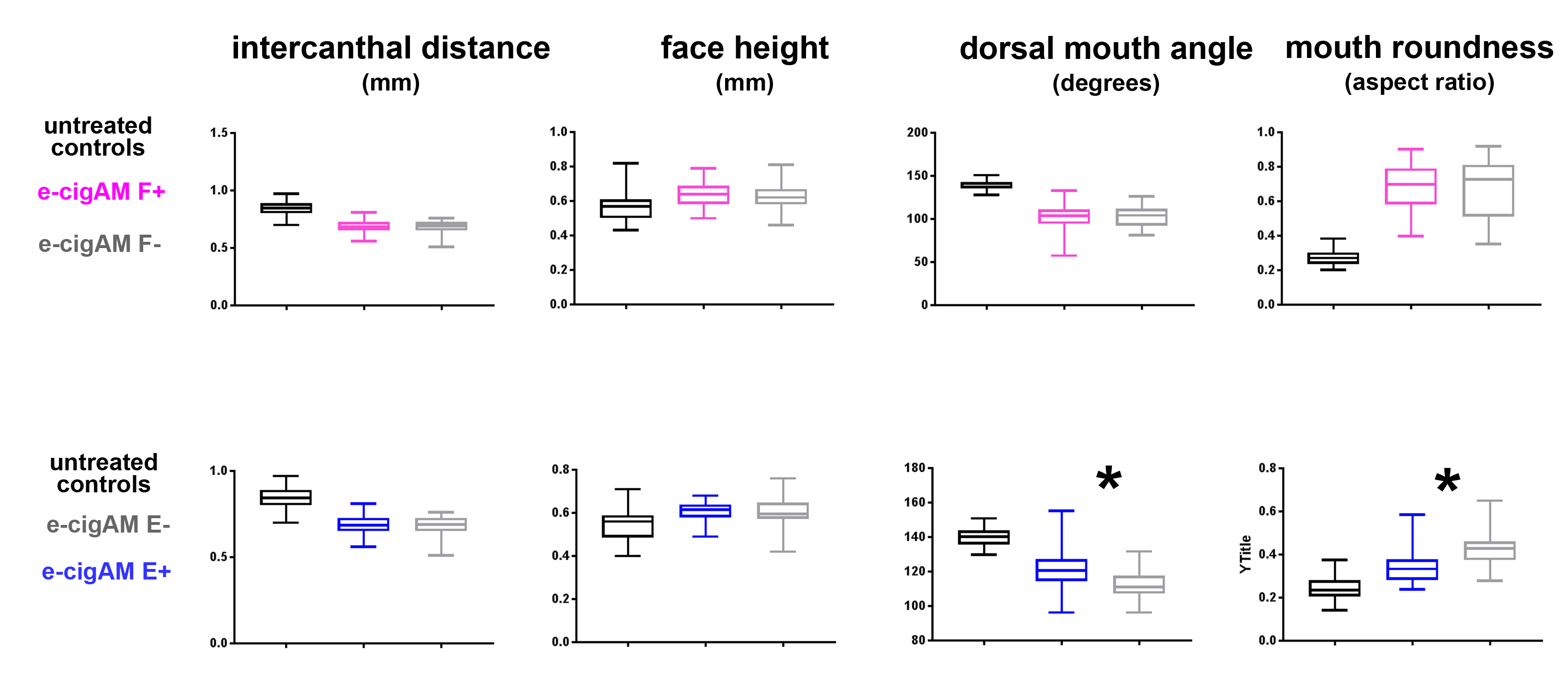

Supplement: S4 Fig — Whisker plots are shown for intercanthal distance, face height, dorsal mouth angle and mouth roundness in the comparison of e-cigAM E and F with and without nicotine. The median is represented by the line through the box, the top whisker represents the maximum value and the bottom whisker represents the minimum value. Asterisks represent statistically significant differences. (TIF) [file pone.0185729.s004.tif]
